# Spatiotemporal regulation of cAMP signaling controls the human trophoblast fusion

**DOI:** 10.3389/fphar.2015.00202

**Published:** 2015-09-15

**Authors:** Pascale Gerbaud, Kjetil Taskén, Guillaume Pidoux

**Affiliations:** ^1^INSERM, UMR-S-1139, Group Cell Fusion, Université Paris DescartesParis, France; ^2^Université Paris DescartesParis, France; ^3^Centre for Molecular Medicine Norway, Nordic EMBL Partnership, University of Oslo and Oslo University HospitalOslo, Norway; ^4^Biotechnology Centre, University of OsloOslo, Norway; ^5^K.G. Jebsen Inflammation Research Centre, University of OsloOslo, Norway; ^6^K.G. Jebsen Centre for Cancer Immunotherapy, University of OsloOslo, Norway; ^7^Department of Infectious Diseases, Oslo University HospitalOslo, Norway; ^8^INSERM, U1180Châtenay-Malabry, France; ^9^Faculté de Pharmacie, Université Paris-SudChâtenay-Malabry, France

**Keywords:** protein kinase A, cAMP, AKAPs, phosphodiesterases, phosphatases, placenta, trophoblast fusion

## Abstract

During human placentation, mononuclear cytotrophoblasts fuse to form multinucleated syncytia ensuring hormonal production and nutrient exchanges between the maternal and fetal circulation. Syncytial formation is essential for the maintenance of pregnancy and for fetal growth. The cAMP signaling pathway is the major route to trigger trophoblast fusion and its activation results in phosphorylation of specific intracellular target proteins, in transcription of fusogenic genes and assembly of macromolecular protein complexes constituting the fusogenic machinery at the plasma membrane. Specificity in cAMP signaling is ensured by generation of localized pools of cAMP controlled by cAMP phosphodiesterases (PDEs) and by discrete spatial and temporal activation of protein kinase A (PKA) in supramolecular signaling clusters inside the cell organized by A-kinase-anchoring proteins (AKAPs) and by organization of signal termination by protein phosphatases (PPs). Here we present original observations on the available components of the cAMP signaling pathway in the human placenta including PKA, PDE, and PP isoforms as well as AKAPs. We continue to discuss the current knowledge of the spatiotemporal regulation of cAMP signaling triggering trophoblast fusion.

## Introduction

Cell fusion processes are essential for fertilization, fetal and placental development, skeletal muscle formation, bone homeostasis and appears to play a role in metastasis (Midgley et al., 1963; Zambonin Zallone et al., 1984; Wakelam, 1985; Oren-Suissa and Podbilewicz, 2007; Lu and Kang, 2009). Cell fusion and syncytia formation involve the mixing of plasma membrane components and cell contents between two or more cells. Although occurring in a variety of biological contexts, different fusion processes share many of the same steps that are tightly regulated in space and time (Pérot et al., 2011).

**FIGURE 1:**
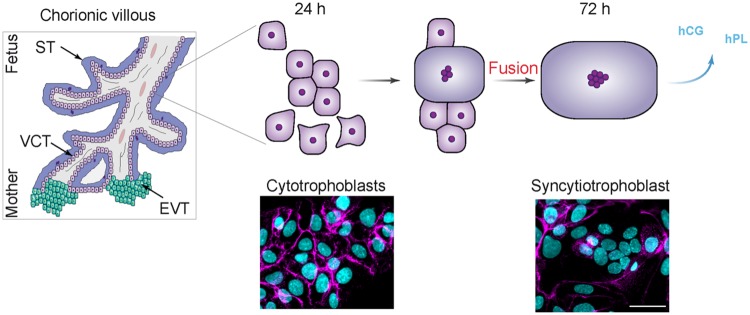
**Model of cultured villous trophobasts purified from human placenta. (Left)** Schematic view of human chorionic villi. VCT for villous cytotrophoblast, ST for syncytiotrophoblast, and EVT for extravillous trophoblast. (**Right**, upper) Model of trophoblast fusion. Cytotrophoblasts (CT) aggregate after 24 to 48 h of culture, and fuse into a ST after 72 h, which secretes human chorionic gonadotropin (hCG) and human placental lactogen (hPL). (**Right**, Lower) Human trophoblast stained at 24 and 72 h of culture for desmoplakin (magenta) and nuclei (DAPI, cyan). Scale bar: 15 μm.

Human embryo implantation requires placentation, a process in which fetal cytotrophoblasts (CT) invade the maternal endometrium to form an interphase with the maternal circulation, ensuring effective exchange of gases, nutrients, and the secretion of pregnancy-specific hormones (i.e., hCG: human chorionic gonadotropin and hPL: human placental lactogen) (Eaton and Contractor, 1993; Ogren and Talamentes, 1994; Benirschke and Kaufmann, 2000). Throughout pregnancy CTs fuse to form a multinucleated syncytia on chorionic villi extending into the maternal placental blood circulation. Due to their capacity to differentiate into syncytia allowing essential placental exchange between mother and child necessary for fetal growth, the CT plays an essential role during human pregnancy. The *in vivo* fusion process in the placenta is reproducible *in vitro* using purified human CTs, which aggregate and then fuse to form non-proliferative, multinucleated, endocrine active syncytiotrophoblasts (STs; **Figure 1**; Kliman et al., 1986). Numerous proteins have been reported to be implicated in cell fusion processes such as tight junction, adherens junction, and gap junction proteins (Coutifaris et al., 1991; Dahl et al., 1995; Mbalaviele et al., 1995; Ilvesaro et al., 2000; Frendo et al., 2003; Charrasse et al., 2007; Pidoux et al., 2010).

**FIGURE 2:**
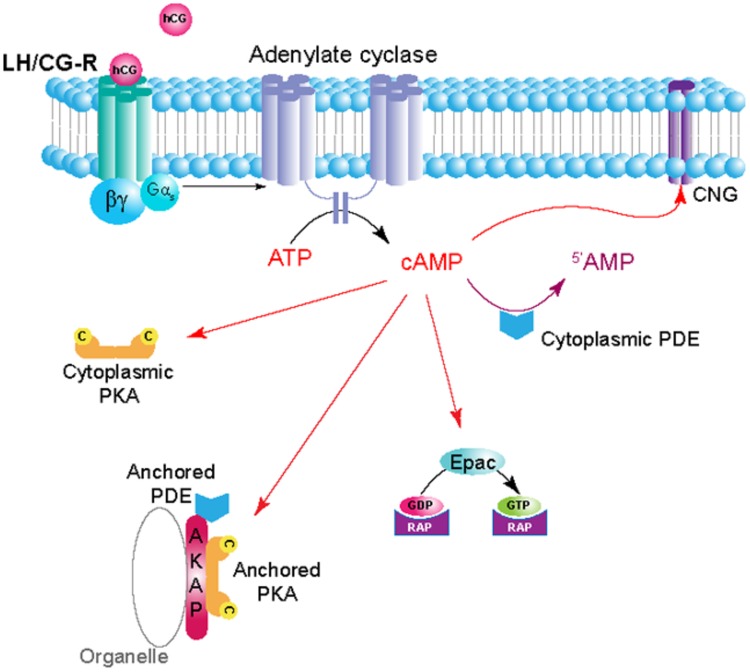
**Schematic depiction of hCG/cAMP signaling pathway in trophoblasts.** Ligand (hCG) binding to the LH/CG-R receptor (LH/CG-R), a G protein-coupled receptor (GPCR), activates adenylyl cyclase (AC) in its proximity and generates pools of cAMP. The local concentration and distribution of the cAMP gradient is limited by phosphodiesterases (PDEs), that hydrolyse cAMP to 5′-AMP. Subcellular structures may harbor specific isozymes of protein kinase A (PKA) that, through anchoring to A-kinase-anchoring proteins (AKAPs), are localized in the vicinity of the receptor and the cyclase. PDEs are also anchored and serve to limit the extension and duration of cAMP gradients. These mechanisms serve to localize and limit the assembly and triggering of specific pathways to a defined area of the cell close to the substrate. Cyclic AMP has effects on a range of effector molecules encompassing PKA, PDEs, Epacs (exchange proteins activated by cAMP) and cyclic nucleotide-gated ion channels (CNGs).

The cAMP signaling pathway is known to play a critical role in induction of CT and myoblast cell fusion reviewed in (Gerbaud and Pidoux, 2015). For instance, in human placentation, hCG stimulates cytrophoblast fusion in an autocrine or paracrine fashion through binding to the LH/CG receptor (LH/CG-R), activating a specific adenylyl cyclase (AC) and the synthesis of intracellular cAMP as second messenger (**Figure 2**). The nine transmembrane AC isoforms have been identified with various expression levels in human CT and on the plasma and microvillous membrane of the ST (Sato and Ryan, 1971; Matsubara et al., 1987; Bernatchez et al., 2003).

Next, the cAMP increase leads to activation of down-stream effectors such as cAMP-dependent protein kinase A (PKA) and the phosphorylation of specific targets (Tasken and Aandahl, 2004). Whereas, PKA is known to be the main cAMP effector, other intracellular effectors exist such as exchange proteins activated by cAMP (Epac) and the cyclic nucleotide-gated ion channels (Walsh et al., 1968; Nakamura and Gold, 1987; de Rooij et al., 1998; Kawasaki et al., 1998) (**Figure 2**). The cAMP signaling pathway is one of the best-characterized signal transduction pathways and requires a high level of spatial and temporal regulation to convey the appropriate inputs. The temporal regulation is achieve by the cAMP synthesis through AC and metabolized by cAMP-phosphodiesterase (PDE) activity. Both AC and PDE establish in cAMP microdomains within the cell (Zaccolo and Pozzan, 2002). Furthermore, A-kinase anchoring proteins (AKAPs) provide through PKA anchoring a spatial regulation of the cAMP/PKA signaling by placing the kinase in the vicinity substrates.

Little is known about AKAP proteins underlying the spatio-temporal regulation of the cAMP-induced human cytrophoblast fusion and regulation of endocrine functions in the placenta. However, we have examined the functional role of AKAP-anchored signaling complexes human primary CTs in a recent report (Pidoux et al., 2014). Here we provide for the first time original data and on the repertoire of cAMP signal components in the human placenta and compile the current knowledge about the role of the AKAP proteins underlying cell fusion process.

## Materials and Methods

### Cell Culture

Villous CTs were isolated from term placentas and cultured as described previously (Pidoux et al., 2014). BeWo cells were obtained from American Type Culture Collection (Manassas, VA, USA) and cultured as described by manufacturer.

### Immunolocalization Studies

Immunocytofluorescence was performed as described previously (Pidoux et al., 2014).

### Fusion Assay

Cell fusion was quantified by trophoblast fusion assays as described previously (Pidoux et al., 2014). Briefly, fusion indices were calculated as the ratio of the number of nuclei in STs divided by the number of total nuclei. A ST was defined as at least three nuclei surrounded by a cell membrane as identified by discontinuous desmoplakin immunostaining.

### Protein Sample Preparation and Immunoblot Analysis

Cell extracts were prepared as described previously (Pidoux et al., 2014). Protein samples were resolved by SDS-PAGE and immunoblotted with antibodies (catalog numbers and supplier are indicated unless stated above) against PKA RIα (0.25 μg/ml; 4D7; BD Biosciences), PKA RIIα (0.25 μg/ml; 612243; BD Biosciences), PKA RIIβ (0.25 μg/ml; 610626; BD Biosciences); PKA Cα/β (0.25 μg/ml; 610980; BD Biosciences); AKAP18 (0.5 μg/ml; WH0009465M1; Sigma–Aldrich); GAPDH (1 μg/ml; G8795; Sigma–Aldrich). After incubation with the appropriate HRP-conjugated secondary antibody, blots were developed by using Supersignal West Pico substrate (Thermo Scientific, Illkirch, France).

### R-Overlays

R-overlays were performed as described previously (Hausken et al., 1998) by using ^32^P-labeled recombinant murine RIIα. Membranes with immobilized proteins were blocked in Blotto (5% (w/v) non-fat dry milk plus 0.1% BSA in TBS). Purified recombinant R (4 μg) was radiolabeled with purified catalytic subunit (C) (0.02 μg/μl) and [γ-^32^P]ATP (1.4 μCi/μl) in a buffer containing 50 mM MOPS pH 6.8 with 50 mM NaCl, 2 mM MgCl_2_, and 1 mM DTT, and separated from free ^32^P-ATP by gel filtration in G-50 sepharose. Membranes were incubated with 1 × 10^6^ cpm/ml of ^32^P-labeled recombinant R in TBS-T. For competition assays, Ht31 peptide was added to the radiolabeled R at a concentration of 500 nM and incubated for 2 h before the membranes were added. The membranes were washed in TBS-T and subjected to autoradiography.

### Peptide Synthesis and Loading

Peptides used in trophoblast fusion assay (PKI: R9-TYADFIASGRTGRRNAI and scrambled PKI: R11-ANITSGYFDTIAAGR) were synthesized on an Intavis MultiPep robot (Intavis Bioanalytical Instruments AG), uncoupled and verified by high performance liquid chromatography (HPLC). The concentrations of the peptides were determined by amino acid analysis using an amino acid analyzer from Thermo Scientific Dionex. PKI peptide and its respective control were used at 10 μM.

### Pull-Down Assays

cAMP pulldown assays (8-AHA-cAMP-agarose beads) were performed as described previously (Pidoux et al., 2014). The lysate-bead suspensions were subjected to LC-MS/MS.

### Protein Identification by LC–MS/MS

Protein identifications were performed as described previously (Pidoux et al., 2014).

### siRNA and Mammalian Expression Vector Transfection

Transfections of siRNA or plasmid were performed using Lipofectamine 2000 CD reagent (Life Technologies). Silencing RNA transfections [performed as described previously (Pidoux et al., 2014)] were performed with AKAP18 stealth siRNA (HSS145157) and control (Life Technologies). Mammalian expression vector (2 μg) was incubated with the cells for 48 h at 37°C. ^T^Epac^V V^ clone was described previously (Klarenbeek et al., 2011). The construct was verified by sequencing. Transfection efficiency was determined to be >45%.

### Optical Biosensor Recordings

Optical recording with biosensor was performed as previously described (Polito et al., 2013). Wide-field images were obtained with Olympus BX50WI upright microscope with a 20× 0.5 NA or a 40× 0.8 NA water-immersion objective and an ORCA-AG camera (Hamamatsu). Images were acquired with iVision (Biovision). The excitation and dichroic filters were D436/20 and 455dcxt. Signals were acquired by alternating the emission filters, HQ480/40 for CFP and D535/40 for YFP, with a filter wheel (Sutter Instruments). Image acquisition was triggered manually, except for kinetics measurement where images were acquired automatically within 3–5 s intervals. Pseudocolor images display the ratio value coded in hue and the fluorescence intensity coded in intensity. A calibration square indicates the intensity values from left to right and the ratio values from bottom to top. The size of the square indicates the scale of the image in microns.

### RNA Extraction

Total RNA was extracted from primary trophoblast cells after 24 or 72 h of culture by using the Trizol reagent (Life Technologies). The yield of extracted RNA was determined by measuring optical density at 260 nm. The purity and quality of extracted RNA were subsequently assessed by electrophoresis on 1% agarose gel with ethidium bromide staining. Only high-integrity RNA samples were used for PCR analysis.

### RT-Polymerase Chain Reaction

Reverse transcription-polymerase chain reaction (RT-PCR) was performed with the TaqMan^®^Array Human Phosphodiesterase (4414075, Applied Biosystem) and Human PrimePCR Phosphatases (H384, Biorad) or primers ordered from Santa-Cruz Biotechnology according to the manufacturers’ protocols for analysis of PDEs, PPs, and AKAPs, respectively. GAPDH or ACTB mRNA and 18S RNA levels were used as endogenous RNA controls and were tested to remain constant during trophoblast fusion. Relative gene expression (ΔC_T_) was calculated by subtracting the signal threshold cycle (C_T_) of 18S or GAPDH from the C_T_ value of each studied gene. Subsequently, ΔΔC_T_ values were calculated by subtracting 18S or GAPDH ΔC_T_ (set as calibrator) from the ΔC_T_ of each individual gene and transformed and presented as 2^-ΔΔCT^ in order to obtain the relative gene expression (fold) of genes of interest.

### Phosphodiesterase Activity

LANCE Ultra cAMP assay was modified and adapted to total cell lysate to measure the cAMP-PDE activity. The europium chelate (Eu)-cAMP tracer molecule is captured by an ULight-labeled anti-cAMP monoclonal antibody (mAb), which brings donor and acceptor dye molecules into close proximity. Following irradiation of the samples at 340 nm, the excited energy of the Eu chelate donor is transferred by FRET to the ULight acceptor dye. Thus ULight molecules emit a signal detectable at 665 nm in TR-FRET mode. Residual energy from the Eu chelate produce light at 615 nm and remains constant. In the absence of free cAMP, maximal TR-FRET signal is achieved. Free cAMP produced by stimulated cells competes with the Eu-cAMP tracer for the binding to the ULight mAb, causing a decrease in TR-FRET signal proportional to the concentration of cAMP produced. LANCE Ultra cAMP provides an assay with S/B ratio of 44.6 and an IC50 of approximately 1 nM.

Proteins from trophoblast cell lysate (20 μg) were incubated in presence of 6 nM cAMP with PDE antagonists or DMSO as vehicle, for 90 min at 24°C and under shaking. Subsequently (Eu)-cAMP tracer molecule and ULight-labeled anti-cAMP antibody were added to the solution prior to analysis. Cyclic AMP hydrolysis was below 20%. Histograms represented the profile of PDE activity in trophoblasts.

### Statistical Analysis

The GraphPad Prism 6 software package was used for statistical analysis. Differences between groups were evaluated with ANOVA. *Post hoc* analysis (Tukey) was used for individual comparisons and to obtain *p*-values shown in the figure legends. The sample size and significance level is shown in the figure legends for each graph. All data are presented as means ± SEM. *P* < 0.05 was considered statistically significant.

## Results

### Protein Kinase A Triggers Human Trophoblasts Fusion

**FIGURE 3:**
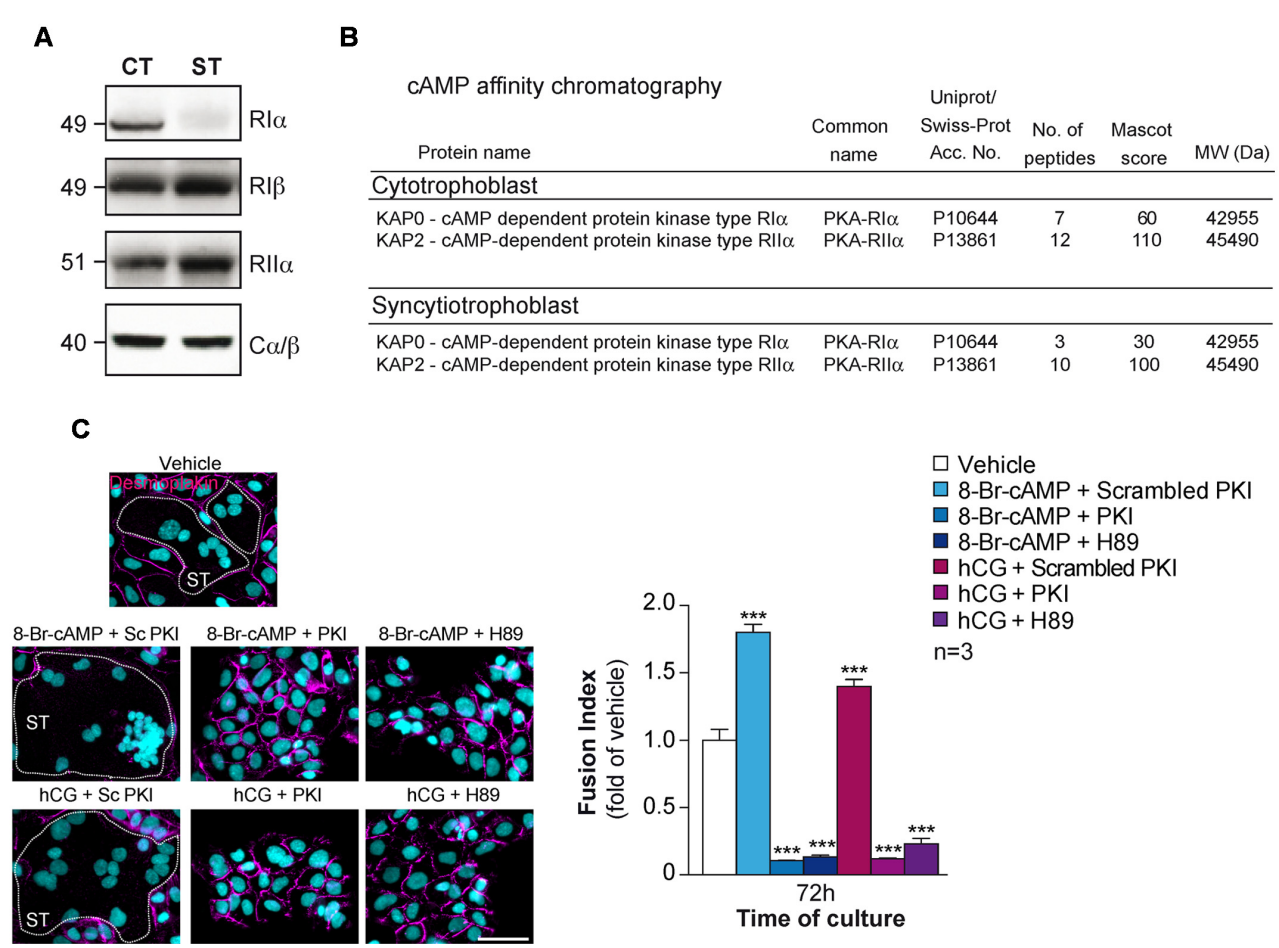
**Characterization of PKA in human trophoblasts. (A)** Immunoblot analysis of RIα, RIβ, RIIα, and Cα/β in lysates of human primary trophoblasts at 24 and 72 h of culture. CT, ST (formed after 72 h of culture). **(B)** PKA R subunits identified by cAMP pulldown in trophoblasts. Proteins from CTs and STs were purified by cAMP affinity chromatography and identified by nanoLC-LTQ Orbitrap Mass Spectrometry analysis of tryptic digests of bands excised from SDS-PAGE. **(C)** Effect of 8-Br-cAMP (100 μM), hCG (1 μM), scrambled PKI or PKI peptide (10 μM each) and H89 (3 μM) on trophoblast fusion at 72 h of culture. Cells were immunostained for desmoplakin (magenta) and nuclei were counterstained with DAPI (left). Syncytia (ST) boundaries are indicated by dashed lines. Effect of 8-Br-cAMP, hCG in combination with PKI peptide or H89 on cell fusion represented as fusion indices histograms (upper right). Results are expressed as the mean ± SEM of *n* = 3 independent experiments (^∗∗∗^*p* < 0.001). Scale bar: 30 μm.

Human placental trophoblasts express RIα, RIIα regulatory subunits and the Cα, Cβ catalytic subunits as evident from immunoblots (**Figures 3A,B**) which support earlier reports (Keryer et al., 1998a,b). RIα is in the cytosol and decreases significantly during the cell fusion process and differentiation to STs while RIIα remains constant and relocates from the cytosol to the Golgi apparatus and to the plasma membrane (Keryer et al., 1998a,b). Interestingly the loss of PKA RIα protein expression during cell fusion process was not associated with a decrease in mRNA level (data not shown), but is probably due to protein destabilization. Indeed, hCG-mediated cAMP increase during syncytialization dissociates and activates PKA holoenzyme and the free RIα and C subunits have been shown in other cell types to be more rapidly degraded to prevent overshoot of catalytic activity in response to signal transduction by cAMP (Tasken et al., 1993). A chemical proteomics approach where we did cAMP affinity chromatography followed by mass spectrometry analysis allowed us to identify PKA RIα and RIIα in human CT and STs (**Figure 3B**), which supported previous reports and our observations by immunoblot analysis. Furthermore, we also observed expression of PKA RIβ isoform in CT and ST at a constant level during cell fusion (**Figure 3A**; Pidoux et al., 2014).

*In vitro*, the addition of cAMP analogs or hCG promote CT fusion to ST, whereas PKA inhibitors (H89) or a cell permeable version of protein kinase inhibitor (PKI) impair cell fusion (**Figure 3C**; Keryer et al., 1998b; Pidoux et al., 2014). It has been proposed that type I PKA is the major PKA holoenzyme that control the cell fusion process and in a less extend type II PKA (Keryer et al., 1998a). Moreover the redistribution of type II PKA during cell fusion is suggested to control the transport of vesicles from *trans*-Golgi network leading to hormone secretion as well as in the reorganization of the subcortical cytoskeleton occurring during the plasmalemma membrane fusion between CT (Keryer et al., 1998a). Interestingly, transient overexpression of the PKA catalytic subunit led to an increase fusion of the trophoblast-derived choriocarcinoma cell line BeWo cells (Knerr et al., 2005).

### A Kinase Anchoring Proteins Control Placental Functions

A kinase anchoring proteins are structurally diverse family of functionally related proteins that include more than 50 members (including space variants) (Pidoux and Tasken, 2010). They are defined on the basis of their ability to bind to PKA and co-precipitate catalytic activity. This binding ensures specific subcellular compartmentalization of the enzyme thereby providing spatial and temporal regulation of the PKA-signaling events. All AKAPs contain a PKA regulatory-binding domain that consists of an amphipatic helix of about 14–18 residues (Carr et al., 1991). Moreover, all AKAPs present a unique targeting domain directing the PKA–AKAP complex to defined subcellular structures, membranes, or organelles (**Figure 2**). In addition to these two domains, several AKAPs are also able to form multivalent signal transduction complexes by interaction with phosphoprotein phosphatases (PPs), kinases, PDEs and other proteins involved in signal transduction (Coghlan et al., 1995; Schillace and Scott, 1999; Feliciello et al., 2001; Tasken et al., 2001; Scott and Pawson, 2009). This scaffolding property of AKAPs functions to coordinate cell-signaling events in space and time and enhance cross talk between signaling pathways. Type II PKA is typically particulate and confined to subcellular structures and compartments anchored by cell- and tissue-specific AKAPs (Colledge and Scott, 1999; Dodge and Scott, 2000; Diviani and Scott, 2001). However, several RI-specific AKAPs have also been characterized (i.e., SKIP, smAKAP), although the type I PKA, which is classically known to be biochemically soluble, has been assumed to be mainly cytoplasmic (Means et al., 2011; Burgers et al., 2012). In addition, dual-specific AKAPs (D-AKAPs) are capable of anchoring both types of R subunits (Huang et al., 1997a,b).

Several AKAPs have been identified in placenta or placental trophoblast cell lines, for instance the AKAP PAP7 is highly expressed in placenta (Li et al., 2001; Liu et al., 2003; Weedon-Fekjær and Taskén, 2012). A number of other AKAPs are expressed in varying amounts in placenta such as AKAP18γ, AKAP350, and AKAP-Lbc 10 kb transcript (Lin et al., 1998; Trotter et al., 1999).

**FIGURE 4:**
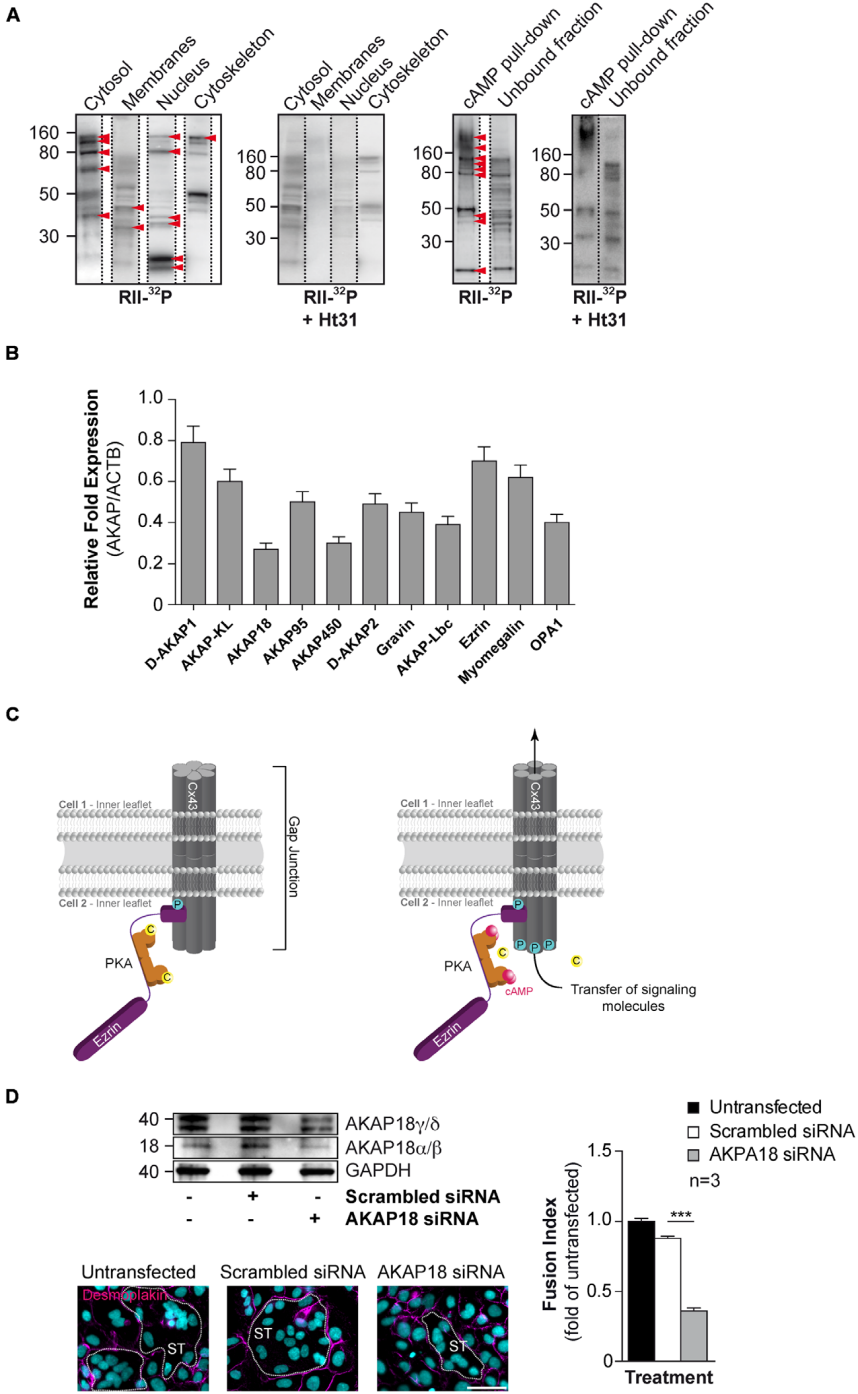
**Characterization of AKAPs in human CTs. (A)** Proteins purified by sub-cellular fractionation (left) or by cAMP affinity chromatography (right) from cytrophoblasts were subjected to a solid phase binding assay using ^32^P-radiolabeled RII (RII-overlay) as a probe in the absence or presence of the Ht31 anchoring disruptor peptide (500 nM). Red arrowheads indicate putative AKAPs expressed in CTs. **(B)** Total RNA was purified from CTs (*n* = 3 cultures each) and subjected to RT-PCR with specific AKAP primers (identified in **Table 1**). Histograms represent mRNA expression of AKAPs in CTs normalized to beta-actin mRNA expression. **(C)** Cx43 gap junction communication is controlled by PKA anchoring through ezrin (Pidoux et al., 2014). Schematic depiction of a resting state gap junction in primary human trophoblast with Cx43 and a compartmentalized pool of PKA anchored to ezrin thus bound to Cx43 (left). Elevated intracellular cAMP levels lead to activation of PKA and subsequent spatiotemporally controlled phosphorylation of Cx43, which promotes the communication through gap junctions. This communication triggers trophoblast cell fusion. C, catalytic subunit of PKA; P for phosphorylation; pink dots, molecules of cAMP. **(D)** BeWo cells were transfected with AKAP18 siRNA (Invitrogen, Cat. # 1299001) or scrambled control, cultured for 48 h, stimulated for fusion with FSK (15 μM) for 24 h and subjected to immunoblot analysis with the indicated antibodies (upper left). Cells with AKAP18 knockdown or controls were stained for desmoplakin (magenta) and nuclei (DAPI). Syncytia (ST) boundaries are indicated by dashed lines. Scale bar: 30 μm. (Lower left) The effects of AKAP18 siRNA, scrambled control on fusion were assessed after 24 h treatment with FSK (15 μM) and summarized in histograms (right). Results are expressed as the mean ± SEM of *n* = 3 independent experiments (^∗∗∗^*p* < 0.001). Scale bar: 30 μm.

In search for AKAPs in primary CTs, proteins from subcellular fractionation or eluates from pull-down of cAMP-binding proteins using 8-AHA-cAMP-agarose beads were separated by SDS-PAGE, blotted to nitrocellulose membranes which were overlaid with radiolabelled RII in the absence or presence of Ht31 anchoring disruptor peptide (**Figure 4A**). Several bands with molecular masses in range of ∼160 and ∼15 kDA were detected in cytosol, membrane, nucleus, and cytoskeleton fraction by RII-overlay and competed by Ht31 peptide (**Figure 4A**). In the same manner, bands with similar molecular masses were detected by RII-overlay following pull-down of cAMP-binding proteins (**Figure 4A**). Our original observations are in agreement with previous studies looking at mRNA expression of AKAPs and suggesting existence of several AKAPs in human primary CTs. To identify AKAPs involved in the cell fusion process, we isolated cAMP-signaling complexes from cultures CTs and ST, either by pull down of cAMP-binding proteins or by FLAG-affinity chromatography after incubation with purified FLAG-tagged regulatory RI and RII subunits, and we subjected bands excised and eluted from SDS-PAGE to tryptic digestion and mass spectrometry analysis. By this approach we identified 11 AKAPs, which are summarized in **Table 1**. An RT-PCR screening strategy was applied to characterize expression of AKAP mRNAs in CTs (**Figure 4B**). We found that these cells expressed AKAP mRNAs with various levels that supports our mass spectrometry analysis. A screen by siRNA-mediated knockdown of various identified-AKAPs allowed us to characterize the involvement of five AKAPs (D-AKAP1, AKAP18, AKAP450, Ezrin, and myomegalin) in the regulation of cell fusion (**Table 1**; Pidoux et al., 2014). This suggests specific roles for pools of AKAP-anchored type I and/or type II PKA in trophoblast fusion.

**Table 1:** A-kinase-anchoring proteins (AKAPs) identified in trophoblasts.

AKAP *(gene nomenclature committee name)*	Method of identification	Subcellular localization	Properties/function	CT/ST	Cell-fusion
D-AKAP1 (*AKAP1*)	RII affinity chromatography	Outer mitochondrial membrane Endoplasmic reticulum Nuclear envelope	Dual-specific AKAP (D-AKAPs) Binds lamin, PP1 and PDE7A Multiple splice variants	CT	Decrease – T
AKAP-KL (*AKAP2*)	RII affinity chromatography	Actin cytoskeleton Apical membrane	Multiple splice variants	CT/ST	No effect – T
AKAP18α, β, γ, δ (*AKAP7*)	cAMP chromatography	Basolateral (α) Apical (β) Plasma membrane (γ) Cytoplasm (γ) Secretory vesicles (δ)	Targeted to plasma membrane Modulation of Na+ Associate to L-type channels (α)	CT	Decrease – BW
AKAP95 (*AKAP8*)	RII affinity chromatography	Nuclear matrix	Chromosome condensation Binds Eg7, condensin and PDE7A	CT/ST	No effect – T
AKAP450 (*AKAP9*)	RII affinity chromatography	Centrosome Golgi	Binds PDE4D3, PP1, PP2A PKN and PKC_𝜀_ Targets PKA and PP1 to NMDA-R Multiple splice variants	CT/ST	Decrease – T
D-AKAP2 (*AKAP10*)	cAMP chromatography	Vesicles Peroxisomes Centrosome	D-AKAPs Binds PP1, PP2A	CT	No effect – T
Gravin (*AKAP12*)	RII affinity chromatography	Actin cytoskeleton Cytoplasm	Binds PKC and β-AR	CT	? – T
AKAP-Lbc (*AKAP13*)	RII affinity chromatography	Cytoplasm	Binds Rho-GEF Couples G_α12_ to Rho	CT/ST	? – T
Ezrin	RI/II and cAMP chromatography	Actin cytoskeleton Plasma membrane	D-AKAPs Binds Cx43 Linked to CFTR via EBP50 RISR domain	CT/ST	Decrease – T
Myomegalin	RII affinity chromatography	Cytoskeleton Centrosome Cytoplasm	Binds PDE4D	CT	Decrease – T
OPA1	RI affinity chromatography	Inner mitochondrial membrane Mitochondrial intermembrane Lipid droplets	D-AKAPs	CT	? –T

*Proteins from cytotrophoblasts and syncytiotrophoblast were purified by cAMP or RI-flag and RII-flag affinity chromatography and identified by nanoLC-LTQ Orbitrap Mass Spectrometry analysis of tryptic digests of bands excised from SDS-PAGE. For each AKAP, the subcellular localization, the properties described in literature and the cellular origin of the identification (CT for cytotrophoblast and/or ST for syncytiotrophoblast) were specified. The effect of tested siRNA mediated knockdown on trophoblast fusion was presented from *in vitro* experiments using primary culture of human trophoblasts (T) or BeWo cells (BW). Silencing of AKAPs not tested on cell fusion was indicated by “?”.*

We recently demonstrated that trophoblast fusion is regulated by ezrin, a known AKAP, which binds to connexin-43 and delivers PKA in the vicinity gap junctions. We found that disruption of the ezrin-Cx43 interaction abolished PKA-dependent phosphorylation of Cx43 as well as gap junction communication and subsequently hCG-induced cell fusion in human primary trophoblasts (**Figure 4C**; Pidoux et al., 2014). This appears to be a general mechanism for gating of Cx43 gap junctions and a dominant mechanism in controlling CT fusion, although not the only mechanism.

Looking at other AKAPs that could be involved in hCG action and cell fusion, AKAP79, AKAP95, and AKAP250 (gravin) were also detected in the trophoblast cell line BeWo (Delidaki et al., 2011). In the same study, the authors demonstrated cross talk between the cAMP/PKA and MAPK pathways involved in secretion of hCG, which acts as an auto-paracrine loop to induce trophoblasts fusion. The authors found that this cross talk was dependent on PKA and PKA–AKAP interaction as a specific PKA inhibitor (myr-PKI) and a PKA anchoring disruptor peptide (Ht31) inhibited the forskolin (FSK)-induced MAPK stimulation (Delidaki et al., 2011). Based on our original observations, siRNA-mediated knockdown of AKAP18 in BeWo cells reduced all isoforms of AKAP18 expression after normalization to GAPDH levels compared with cells transfected with scrambled siRNA (**Figure 4D**). SiRNA mediated depletion of AKAP18 decreased BeWo cell fusion after treatment with FSK (a potent cAMP activator) by approximately 60% (*p* < 0.001) compared to scrambled control transfected cells. It is known that AKAP18 isoforms are involved in the regulation of intracellular calcium fluxes. AKAP18α/β is associated with the plasma membrane and is necessary for PKA-dependent phosphorylation of the L-type Ca^2+^ channel under β-adrenergic stimulation in cardiomyocytes. This phosphorylation by PKA increases the opening probability and conductance of this channel (Fraser et al., 1998). AKAP18γ/δ located to the sarcoplasmic reticulum membrane facilitates adrenergic regulation of calcium reabsorption from cytosol by regulating phospholamban that controls the sarcoplasmic reticulum calcium ATPase Serca2 (Lygren et al., 2007). It is noteworthy that trophoblastic syncytialization requires extracellular calcium (Dimitrov et al., 1993; Rote, 2005). Furthermore, calcium entry through L-type Ca^2+^ channel controls hCG released, which could indirectly triggers cell fusion in an auto-paracrine manner (Meuris et al., 1994). However, more experiments are needed to fully decipher the putative regulation of a PKA–AKAP18 complex in cAMP signaling-induced calcium homeostasis in BeWo cell fusion. Another AKAP, AKAP-121 co-immunoprecipitates with PKA and protein tyrosine phosphatase (PTP) D1 in ST mitochondrial membrane that could be involved in progesterone synthesis (Gomez-Concha et al., 2011).

### Cyclic AMP PDE Control Human Trophoblasts Fusion

Cyclic AMP-PDE contribute to the regulation of local cAMP gradients by specifically hydrolyzing the phosphodiester bond of cyclic nucleotides thereby controlling the cAMP cellular level, mediating its return to the basal state concomitantly forming intracellular AMP (**Figure 2**). The termination of cAMP signaling is conferred by a large enzyme superfamily that includes over 40 different PDE isoforms (Baillie et al., 2005; Lugnier, 2006). In mammals, 3 of the 11 PDE families selectively hydrolyze cAMP (PDEs 4, 7, and 8), three families are selective for cGMP (PDEs 5, 6, and 9), and five families hydrolyze both cyclic nucleotides with varying efficiencies (PDEs 1, 2, 3, 10, and 11) (Lugnier, 2006; Conti et al., 2007). An explanation of the complexity of PDEs and the existence of numerous splicing variants is that the divergent domains specify protein–protein interactions. These interactions engender a catalytic domain with new regulatory properties and integrate the function of the holoenzymes in macromolecular complexes strategically located within the cell. Although PKA may interact directly with some PDEs, most interactions with kinases and phosphatases are mediated by AKAPs and participate to intracellular signaling compartmentation (**Figure 2**; Dell’Acqua and Scott, 1997).

**FIGURE 5:**
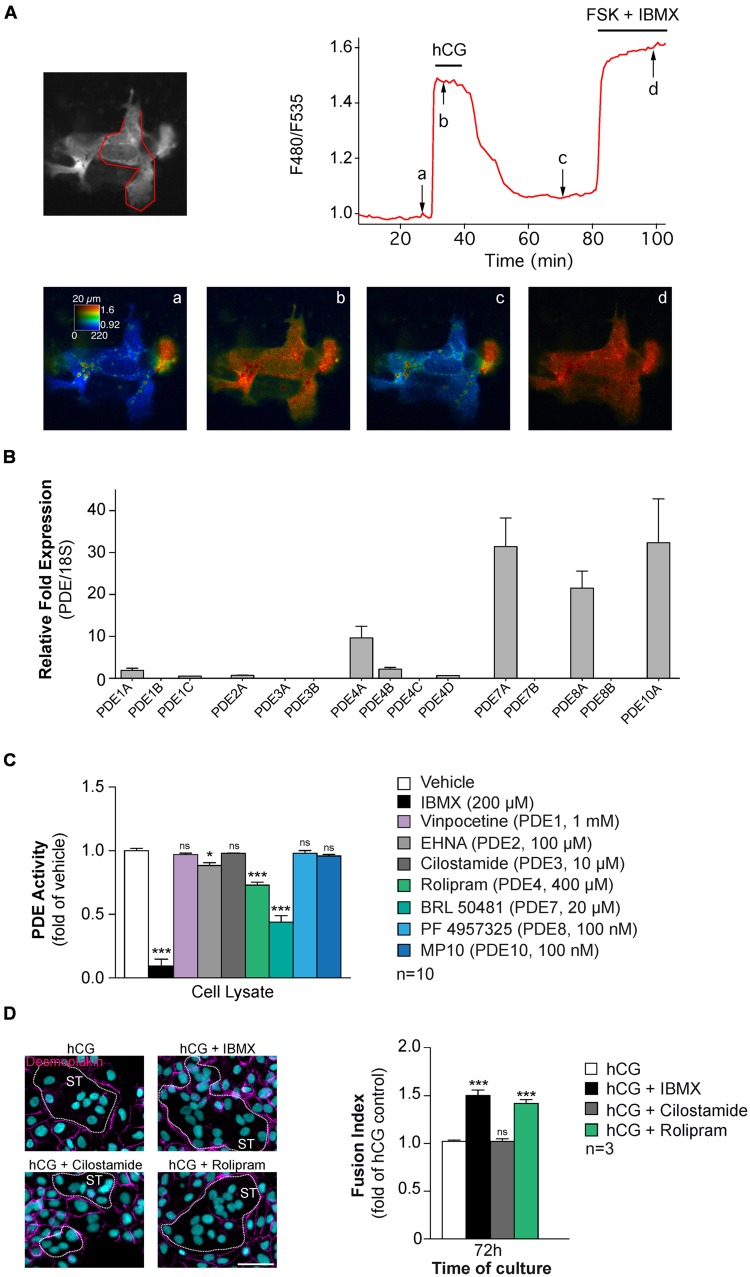
**Characterization of cAMP-PDEs in human CTs. (A)** CTs purified from human placenta were transfected with a DNA construct directing the expression of the ^T^Epac^V V^ biosensor and imaged with wide field microscopy. Images show the fluorescence at 535 nm (upper left, in gray scale) and the ratio (in pseudocolor; lower row) indicating a ratiometric change of ^T^Epac^V V^, reporting the binding of cAMP at the times indicated by arrows on the graph (upper right panel). The trace on the graph indicates the F480/F535 emission ratio measurement on region indicated in red on the gray scale image. Human CG (1 μM) induced a robust ratio increase in CTs. FSK + IBMX incubation were used to induced the maximal response. Data are representative of three separate experiments **(B)** cAMP-PDE mRNA expression profile in CTs. Total RNA was purified from CTs (*n* = 5 cultures) and subjected to RT-qPCR, using Human PDE TaqMan Gene Expression Assays chip (Applied Biosystems). Histograms represent mRNA expression of cAMP-PDEs in trophoblast normalized to 18S rRNA expression. **(C)** Cyclic AMP-PDE activity in human CTs. LANCE Ultra cAMP assay (Perkin Elmer) was adapted to quantify PDE activity in total CT lysates. Trophoblast cell lysates (20 μg) were incubated in presence of 6 nM cAMP with or without PDE antagonists, subsequently (Eu)-cAMP tracer and ULight-labeled anti-cAMP antibody were added to the solution prior to analysis. Histograms represented the profile of PDE activity in trophoblasts. **(D)** Effect of PDE inhibitor on CT fusion. Cytrophoblasts were stimulated with hCG (1 μM) and treated with pan-PDE (IBMX, 200 μM), PDE4 (Rolipram, 4 μM) or PDE3 (Cilostamide, 100 nM) antagonists. Cells were stained for desmoplakin (magenta) and nuclei (DAPI, left), Syncytia (ST) boundaries are indicated by dashed lines and fusion indices were measured (right histograms). Results are expressed as the mean ± SEM of indicated independent experiments (n.s., non significant, ^∗^*p* < 0.05, ^∗∗∗^*p* < 0.001). Scale bar: 30 μm.

The presence of cAMP-PDE activity in human placenta was first reported (Ferre et al., 1975). Since then PDE3 and PDE4 were characterized in cytosolic fraction of human placenta biopsies (Xiong et al., 1990). To further examine the role of PDEs in human primary trophoblast, we performed live-cell imaging and fluorescence resonance energy transfer using ^T^Epac^V V^ biosensor (**Figure 5A**) (Klarenbeek et al., 2011). Trophoblasts were transfected with a plasmid directing the expression of ^T^Epac^V V^ biosensor to visualize the dynamic of cAMP accumulation and PDE activity under hCG stimulation (**Figure 5A**). Activation of the LH/CG-R with a supraphysiological concentration of hCG (1 μM) increased the F480/F535 emission ratio (**Figure 5A**). The maximal response was obtained by the addition of the AC activator FSK (15 μM) in combination with IBMX (pan-PDE inhibitor; 200 μM). These original findings indicate that hCG promotes cAMP synthesis and suggest that PDEs could regulate the cAMP signaling in human CTs. Thus an RT-qPCR screening strategy was applied to characterize PDE genes expression in CTs and ST (**Figure 5B**). Interestingly, the fusion process did not induce changes in the PDE gene expression profile (unpublished data). However, we found that trophoblasts expressed mRNA of PDE7A, PDE8A, and PDE10A isoforms at high levels and PDE4A mRNA to a lesser extent whereas trophoblasts displayed low levels of expression of PDE1A, PDE1C, PDE2A, PDE4B, and PDE4D mRNA. Interestingly, no mRNA expression was observed for PDE1B, PDE3A, PDE3B, and PDE4C isoforms in human trophoblasts. It is noteworthy that PDE3 activity identified by Xiong et al. (1990) was assessed on human placental biopsies comprising other cell types than trophoblasts that may express PDE3. To support these data, we also examined cAMP-PDE activity in human CT cell extracts by using a TR-FRET strategy (**Figure 5C**). As evident from the data, IBMX significantly reduced the PDE activity in trophoblast cell lysates compared to vehicle. Furthermore, using specific antagonists we found mainly PDE4 and PDE7 activity and to a less extent PDE2 activity. Interestingly, no PDE8 and PDE10 activity were detected in CT lysate, whereas mRNA of these two PDE isoforms were characterized. Moreover, no PDE3 activity was found, which is in agreement with our RT-qPCR analysis and the absence of PDE3 mRNA. In order to establish the temporal effect of PDE antagonists on cell fusion of primary trophoblasts from human placenta, cells were cultured for 24 h in the presence of hCG to induce cell fusion with or without IBMX, Cilostamide (PDE3 antagonist), and Rolipram (PDE4 antagonist) (**Figure 5D**). As evident from immunostaining and activity data, the combination of hCG and pan-PDE or PDE4 antagonists potentiated human trophoblast fusion in the same manner compared to cells treated with hCG alone. PDE3 antagonists displayed no effects on cell fusion, which is in agreement with the absence of PDE3 mRNA and activity in human CTs. We demonstrate for the first time that various cAMP-PDEs are expressed in human CT and PDE4 isoform regulates the CT cell fusion. However, more experiments need to be performed to identify the specific PDE4 isoforms that regulates the human trophoblast fusion and in which subcellular compartment this occurs.

### Serine/Threonine PPs Expression Profile in the Primary Human Trophoblasts

**FIGURE 6:**
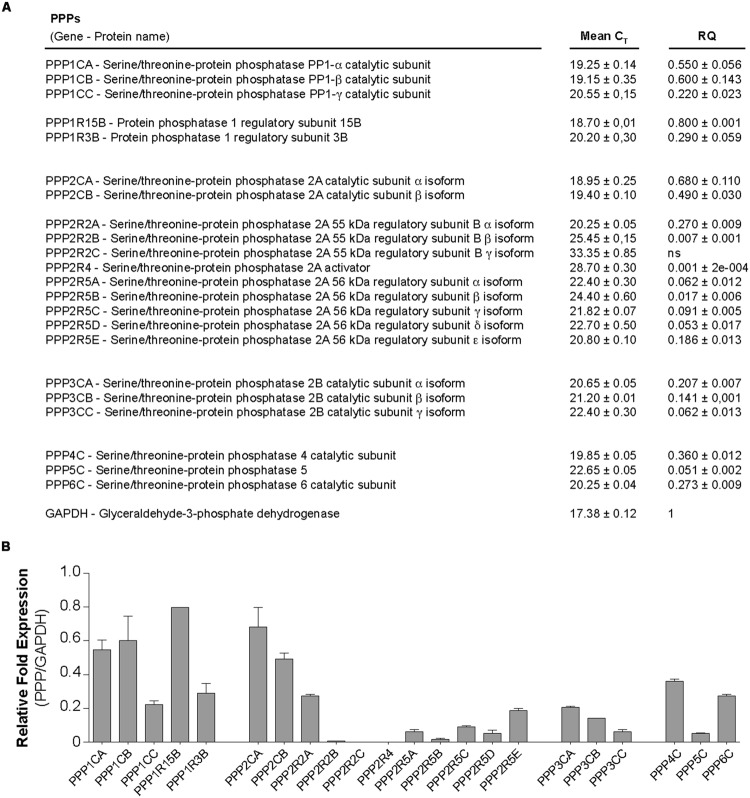
**Characterization of PPs in human trophoblasts.** Messenger RNA expression profile of serine/threonine protein phosphatases (PPs) in trophoblasts. Total RNA was purified from CTs and ST (*n* = 5 cultures each), pooled and subjected to RT-qPCR, using Human Phosphatase Collection Panel chip (Prime PCR, Biorad). **(A)** Table shows mean ± SEM of C_T_ and associated-relative quantification (RQ). **(B)** Histograms represent mRNA expression of PPPs in trophoblast normalized to GAPDH mRNA expression.

To our knowledge, there are no reported studies of PPs in trophoblasts and their putative role in the regulation of cell fusion. An RT-qPCR screening strategy was applied to characterize expression of PP mRNAs in CTs and ST (**Figure 6**). We found that trophoblasts expressed mRNA of PP1, PP2A catalytic subunits, and PP1 regulatory subunit 15B and 3B isoforms and PP2A regulatory subunit alpha isoform (**Figure 6**), whereas expression of calcineurin (PP2B) mRNA was weaker. Interestingly, the fusion process did not induce modifications in PP genes expression profiles (unpublished data). By immunoblotting we characterized expression of PP1-Cα, PP2A-Cα/β, and Calcineurin A subunits in primary human trophoblasts (data not shown). Protein phospho- and dephosphorylation, controlled by kinases and phosphatases, respectively, affect many cellular processes and their regulations must be specific to act on a defined subset of cellular targets to ensure signal fidelity. More experiments need to be done here to identify subcellular targets of PPs and may characterize in detail their functions in the regulation of human trophoblasts fusion.

## Discussion

Trophoblast fusion and ST formation is a complex biological process essential for the maintenance of pregnancy and for fetal growth. For more than two decades, it have been recognized that the cAMP/PKA signaling pathway is the major signaling pathway involved in this process, which modulates the expression of proteins that trigger human trophoblast fusion (Gerbaud and Pidoux, 2015). However, cAMP-induced trophoblast fusion remains a field where a number of molecular details remain to be elucidated. To this end, the characterization of components of the cAMP signaling pathway (PDEs, PPs, and AKAPs) as reported here and may help understand how cAMP signaling as propagated intracellularly to regulate this complex biological process.

In an autocrine–paracrine loop, hCG binds to LH/CG-R at the membrane of human trophoblasts and induces intracellular cAMP production (Shi et al., 1993; Keryer et al., 1998b; Pidoux et al., 2007a,b, 2014). Subsequently, cell fusion is triggered by activation of PKA that phosphorylates the CREB transcription factor (cAMP Response Element-Binding Protein), which associates with CBP (CREB-binding protein) and P300 to increase specific gene expression of fusogenic proteins (i.e., hCG, GCM1, Cx43, and syncytins; Knerr et al., 2005; Chen et al., 2008; Chen and Cheong, 2011; Ul Hussain, 2014). Moreover, induction of cAMP/PKA signaling promotes association between the transcription factors GCM1 and CBP, thereby enhancing the transcription of fusogenic genes such as those encoding Cx43 and syncytins (Chen and Cheong, 2011; Dunk et al., 2012; Gerbaud and Pidoux, 2015). Despite all of these characterized events, specific roles of individual PKA isoforms in the regulation of the trophoblast fusion process remains to be elucidated. Furthermore, precise spatial and temporal activation of PKA inside the cell is accomplished by sequestering PKA in specific locations through interaction with AKAPs and by generation of localized pools of cAMP.

Protein kinase A is considered as the major effector of the cAMP-signaling pathway. However, other intracellular effectors for this signaling exist such as exchange proteins activated by cAMP (Epac) and the cyclic nucleotide-gated ion channels (CNG) (**Figure 2**). CNGs are heterotetrameric complexes that are composed by different types of subunits. CNG channels are non-selective cation channels allowing passage of alkali ions as well as divalent cations (i.e., Ca^2+^) (Kaupp and Seifert, 2002). The opening of CNG channels is under dependence of cyclic nucleotide binding (either cAMP and/or cGMP). To our knowledge, the role and full characterization of CNG channels in trophoblasts and during cell-fusion has not been assessed. Epac have been identified less than two decades ago and characterized as an important effector of the cAMP signaling cascade that act differently that PKA (de Rooij et al., 1998; Kawasaki et al., 1998). Epac signaling is involved in cell differentiation, secretion of vesicles, cell adhesion, and cell–cell adhesion (Bos, 2006). The Epac family comprises Epac1 and Epac2. Epac1 is ubiquitously expressed while Epac2 has been originally identified in brain and adrenal glands (Kawasaki et al., 1998). Epac binds cAMP on a CNB domain (cyclic nucleotide-binding) and subsequently function as a guanine nucleotide exchange factors (GEFs) for Rap1 and Rap2, which belongs to Ras family of small G protein. In the guanosine diphosphate (GDP)-bound state Epac is inactive and becomes active once GDP is exchanged for guanosine triphosphate (GTP). GEFs induce the transfer of GDP for GTP and thereby the activation of the small G protein, whereas GTPase-activating proteins (GAPs) hydrolyze GTP (for review, Gloerich and Bos, 2010). Recent studies undertook to characterize the role of Epac in trophoblast cell fusion. Yoshie et al. (2010) found the expression of Epac1 and Epac2 in CT and STs. In the same report, the authors showed that Epac activation promotes BeWo cell fusion. However, the molecular mechanisms underlying this process were not clear. In another recent report, it has been shown that an Epac/CaMKI signaling cascade works in synergy with PKA signaling to trigger BeWo cell fusion. This cascade activates GCM1 transcription factor, which enhances the transcription of fusogenic genes (Chang et al., 2011). More studies are needed to fully elucidate the molecular mechanisms underlying trophoblast cell fusion and ST regeneration, and thus the pathophysiology of human placental development. Any alteration of syncytial formation and regeneration during pregnancy will affect fetal growth and the outcome of the pregnancy. Anomalies of villous trophoblast differentiation and cell fusion can lead to severe placental abnormalities that could lead to intrauterine growth restriction (IUGR) and preeclampsia (Huppertz and Kingdom, 2004). A better understanding in the cAMP signaling pathway and its regulation in space and time that induce cell fusion could lead to the development of therapeutic tools that may counteract pathologies of the pregnancy with a defect in syncytial formation.

## Author Contributions

GP and KT wrote the paper. GP did artistic work. PG did experiments and commented on the text and figures; all the authors read and commented on the drafts and approved the final version.

## Conflict of Interest Statement

The authors declare that the research was conducted in the absence of any commercial or financial relationships that could be construed as a potential conflict of interest.
